# Alterations of amplitude of low-frequency fluctuations and fractional amplitude of low-frequency fluctuations in end-stage renal disease on maintenance dialysis: An activation likelihood estimation meta-analysis

**DOI:** 10.3389/fnhum.2022.1040553

**Published:** 2022-12-01

**Authors:** Huiling Cao, Feng Lin, Ben Ke, Jianling Song, Yuting Xue, Xiangdong Fang, Erming Zeng

**Affiliations:** ^1^Department of Nephrology, The Second Affiliated Hospital of Nanchang University, Nanchang, Jiangxi, China; ^2^Department of Neurosurgery, The First Affiliated Hospital of Nanchang University, Nanchang, Jiangxi, China

**Keywords:** end-stage renal disease, dialysis, amplitude of low-frequency fluctuation, fractional amplitude of low-frequency fluctuation, activation likelihood estimation

## Abstract

**Background:**

Cognitive impairment (CI) is a common complication of end-stage renal disease (ESRD). Many resting-state functional magnetic resonance imaging (rs-fMRI) studies have identified abnormal spontaneous low-frequency brain activity in ESRD dialysis patients. However, these studies have reported inconsistent results. So far, no meta-analyses on this topic have been published. This meta-analysis aimed to identify the more consistently vulnerable brain regions in ESRD patients at rest and to reveal its possible neuropathophysiological mechanisms.

**Methods:**

We systematically searched PubMed, Cochrane Library, Web of Science, Medline, and EMBASE databases up to July 20, 2022 based on the amplitude of low-frequency fluctuation (ALFF) or fractional amplitude of low-frequency fluctuation (fALFF). Brain regions with abnormal spontaneous neural activity in ESRD compared to healthy controls (HCs) from previous studies were integrated and analyzed using an activation likelihood estimation (ALE) method. Jackknife sensitivity analysis was carried out to assess the reproducibility of the results.

**Results:**

In total, 11 studies (380 patients and 351 HCs) were included in the final analysis. According to the results of the meta-analysis, compared with HCs, ESRD patients had decreased ALFF/fALFF in the right precuneus, right cuneus, and left superior temporal gyrus (STG), while no brain regions with increased brain activity were identified. Jackknife sensitivity analysis showed that our results were highly reliable.

**Conclusion:**

Compared to HCs, ESRD dialysis patients exhibit significant abnormalities in spontaneous neural activity associated with CI, occurring primarily in the default mode network, visual recognition network (VRN), and executive control network (ECN). This contributes to the understanding of its pathophysiological mechanisms.

**Systematic review registration:**

[https://www.crd.york.ac.uk/prospero/], identifier [CRD42022348694].

## Introduction

A common comorbidity of end-stage renal disease (ESRD) is cognitive impairment (CI). CI is a deterioration of cognitive function beyond that expected with normal aging and is usually acquired and progressive (Busse et al., 2006). Poorer cognitive function may reduce quality of survival and treatment adherence, increase health care costs, morbidity, and mortality (van Zwieten et al., 2019). Many studies have shown that the prevalence of CI is higher in patients with chronic kidney disease (CKD) than in the general population and is more common in patients with ESRD, including varying degrees of impairment in perception, memory, attention, and motor function (O’Lone et al., 2016; Viggiano et al., 2020). According to O’Lone et al. (2016), up to 70% of patients on dialysis have moderate to severe CI at no less than 55 years of age, three times more than controls of the same age (Griva et al., 2010). Currently, the pathogenesis of CI in ESRD patients is unclear. Moreover, its diagnosis is mainly based on clinical symptoms, neuropsychological assessments and neuroimaging examinations, and there is a lack of reliable and objective biological markers. Therefore, it is particularly important to explore the exact pathophysiological mechanisms.

In previous studies, ESRD patients had poorer cognitive test scores compared to the general population (Luo et al., 2016). Neuroimaging studies had found alterations in brain structure and local neurological function in ESRD. For example, previous magnetic resonance imaging (MRI) studies had found that ESRD patients presented with more severe brain atrophy (Drew et al., 2013), significantly reduced brain gray matter volume (Qiu et al., 2014), cortical thinning (Chiu et al., 2019; Richerson et al., 2021), and decreased white matter integrity (Hsieh et al., 2009; Chiu et al., 2019) compared to healthy controls (HCs), all of which are associated with CI (Zhang et al., 2013). In addition, different analyses such as positron emission tomography (PET) (Kuwabara et al., 2002) and arterial spin-labeled (ASL) imaging (Jiang et al., 2016) had also identified hemodynamic, metabolic, and functional alterations in specific brain regions in ESRD patients.

In recent years, functional MRI has developed rapidly and has become an important aid in diagnosing brain function. Task-state functional MRI has differences in experimental design, patient cooperation, and the task itself, which can easily lead to heterogeneity (Bennett and Miller, 2010; Pan et al., 2017a). Resting-state functional magnetic resonance imaging (rs-fMRI) is widely used to explore brain function in patients with neuropsychiatric disorders due to its non-invasive and task-free features, providing a new perspective on the pathophysiological mechanisms of CI in ESRD patients (Wang et al., 2020). The amplitude of low-frequency fluctuation (ALFF) or fractional amplitude of low-frequency fluctuation (fALFF) is a common data analysis method for rs-fMRI and is commonly used to detect spontaneous brain activity when subjects are not performing a task. The ALFF is a measure of blood oxygen level-dependent (BOLD) signal fluctuations in a specific low frequency range (0.01–0.08 Hz) based on voxel levels, which can respond to the strength of local neuronal activity (Yang et al., 2007). The fALFF serves as a standardized index of ALFF, which reduces the effect of physiological noise and has higher sensitivity and specificity in detecting spontaneous brain activity on low-frequency amplitude signals of neurons, but is less reliable than ALFF (Zou et al., 2008). Nowadays, rs-fMRI is used in diseases such as Alzheimer’s disease (Ibrahim et al., 2021; Zhang et al., 2021), depression (Gong et al., 2020), Parkinson’s disease (Jia et al., 2018), and epilepsy (Zhu et al., 2021). Several previous studies have used ALFF/fALFF to explore the pathophysiological mechanisms of ESRD and have found abnormal spontaneous brain activity in a wide range of brain regions. These include precuneus (Luo et al., 2016; Li et al., 2018; Chen et al., 2020; Peng et al., 2021; Su et al., 2021), parietal lobe (Luo et al., 2016; Chen et al., 2020; Peng et al., 2021), posterior cingulate cortex (Luo et al., 2016; Peng et al., 2021), medial prefrontal cortex (Luo et al., 2016; Li et al., 2018), occipital lobe (Luo et al., 2016; Peng et al., 2021), angular gyrus (Chen et al., 2020; Peng et al., 2021; Su et al., 2021), cuneus (Chen et al., 2020; Peng et al., 2021; Su et al., 2021), and others. However, these findings report a wide variety of brain regions.

Considering the above findings of ALFF/fALFF and the inconsistent results of the studies, we conducted a study using the most common meta-analysis method in the field of brain imaging, ALE meta-analysis, aiming to identify reliable neuroimaging markers and possible pathophysiological mechanisms by performing a comprehensive analysis of abnormal brain regions in ESRD patients identified in previous studies using ALFF/fALFF methods.

## Materials and methods

Our study followed the meta-analysis of observational studies in epidemiological (MOOSE) guideline (Stroup et al., 2000) and had been pre-registered in the PROSPERO database (CRD42022348694).

### Literature search and study selection

A comprehensive search of MEDLINE, Cochrane Library, PubMed, Web of Science, and EMBASE databases for studies from inception until July 2022 that reported altered brain activation in patients with ESRD using the ALFF/fALFF method was conducted. The following keywords were used: (“resting-state functional magnetic resonance imaging” OR “rs-fMRI” OR “amplitude of low-frequency fluctuation” OR “ALFF” OR “fractional amplitude of low-frequency fluctuation” OR “fALFF”); AND “cognitive impairment” or “CI”; AND “end-stage renal disease” OR “ESRD” OR “dialysis”. To prevent omissions, the citations of review articles and included studies were manually searched to identify additional articles.

Studies were included in this meta-analysis if they (1) met the diagnostic criteria for ESRD (Levey et al., 2005); (2) maintained on dialysis for ≥3 months; (3) compared the ALFF/fALFF between ESRD and HCs; (4) reported three-dimensional coordinates [Montreal Neurological Institute (MNI) or Talairach] of the whole brain. This meta-analysis excluded studies with the following conditions: (1) age < 18 years; (2) research based on other disorders such as depression, schizophrenia, etc., and (3) review articles, case reports, letters, conference abstracts, and editorials. Two reviewers (HC and FL) independently completed the literature search and screening process to determine the final inclusion in the meta-analysis. Where there were disagreements between reviewers, these were resolved by a consensus reviewer (XF).

### Date extraction

The two reviewers (HC and FL) independently extracted and summarized the required information from the included studies. Data collected include study and subject characteristics [name of the first author, year of publication, the sample size of subjects, mean age, male/female ratio, duration of dialysis, education level, and Montreal Cognitive Assessment (MoCA) scores], imaging characteristics [method of analysis, peak coordinates of activated brain regions (Foci), three-dimensional coordinates (MNI/Talairach), scanner field strength and analysis software].

### Assessment of methodological quality

Independent reviewers (HC and FL) used the Newcastle-Ottawa scale (NOS) (Stang, 2010) to assess the quality and risk of bias of the included literature. The NOS has three dimensions (choice, comparability, and exposure), with eight entries and a total score of nine. If there were differences between reviewers, consensus reviewers (XF) would discuss them together to resolve these differences.

### Data analysis

A total of 100 foci were reported in 11 trials involving 671 participants in this meta-analysis. The results of comparing ESRD and HCs were divided into two groups based on increased and decreased ALFF/fALFF: (a) ESRD: increased ALFF/fALFF (5 experiments; 10 foci); (b) ESRD: decreased ALFF/fALFF (11 experiments; 90 foci). This study followed the latest recommendations for ALE meta-analysis using GingerALE version 3.0.2^1^ (Müller et al., 2018). The ALE method used activation likelihood as an indicator to calculate the likelihood of activation across experiments for each voxel and to test hypotheses on these likelihoods to obtain consistency of brain activation across multiple experiments. First, the stereotactic coordinates (X, Y, and Z) of the studies included in the meta-analysis were extracted, and the extracted coordinates were the brain coordinates that underwent changes in dialysis ESRD compared to HCs. To ensure that all coordinates were in the same coordinate system when analyzed, all Talairach coordinates were converted to MNI coordinates using the GingerALE converter foci tool (Lancaster et al., 2007). Then, all the foci and basic information were sorted into two text files. These files were imported into the software to read the foci. According to the recommendations of Müller et al. (2018), cluster-level corrected family-wise error (FWE) at *p* < 0.05, cluster-forming threshold at *p* < 0.001, and 5,000 permutations were used to calculate the ALE diagram. To better present the results of the result, we used the Mango software^2^ to present the results into a standard template (Colin27_T1_seg_MNI) (Lancaster et al., 2010).

In addition, Jackknife sensitive analysis was used to assess the robustness of the results of the meta-analysis, excluding one dataset at a time (Lyles and Lin, 2010; Pan et al., 2017b). After excluding one study at a time, the data from the remaining studies were subjected to a repeated ALE meta-analysis using GingerALE 3.0.2 software. In general, we considered the result to be highly replicable if a brain region remained significant in most (>50%) of the study combinations (Eickhoff et al., 2016; Pan et al., 2017b).

## Results

### Search results

**FIGURE 1:**
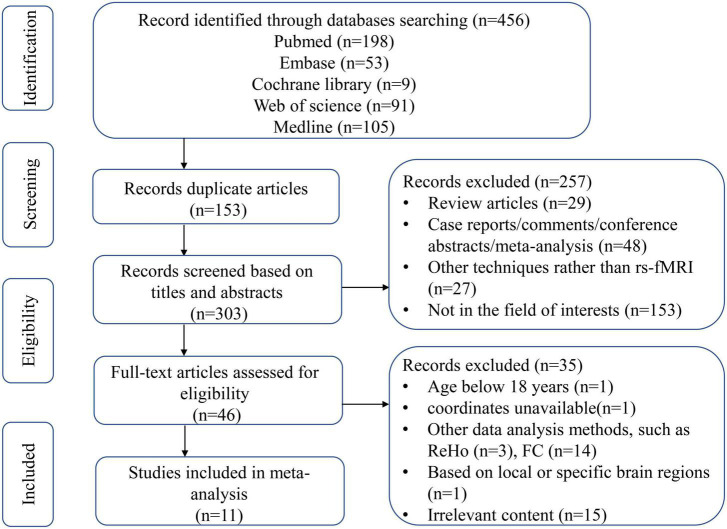
Flow diagram of article selection. ReHo, regional homogeneity; FC, functional connectivity.

Figure 1 showed the literature screening process and results. In total, 456 studies were identified by the search strategy described, 153 studies were excluded because of duplication, and 257 studies were excluded for titles and abstracts. We screened the remaining 46 studies and further excluded 35 studies for the following reasons: not in the area of interest (*n* = 15), age < 18 years (*n* = 1), coordinates unavailable (*n* = 1), based on local or specific brain regions (*n* = 1), other data analysis methods, such as regional homogeneity (ReHo) (*n* = 3) and functional connectivity (FC) (*n* = 14). Finally, 11 studies including 671 subjects were included in the meta-analysis.

### Characteristics and quality assessment

**FIGURE 2:**
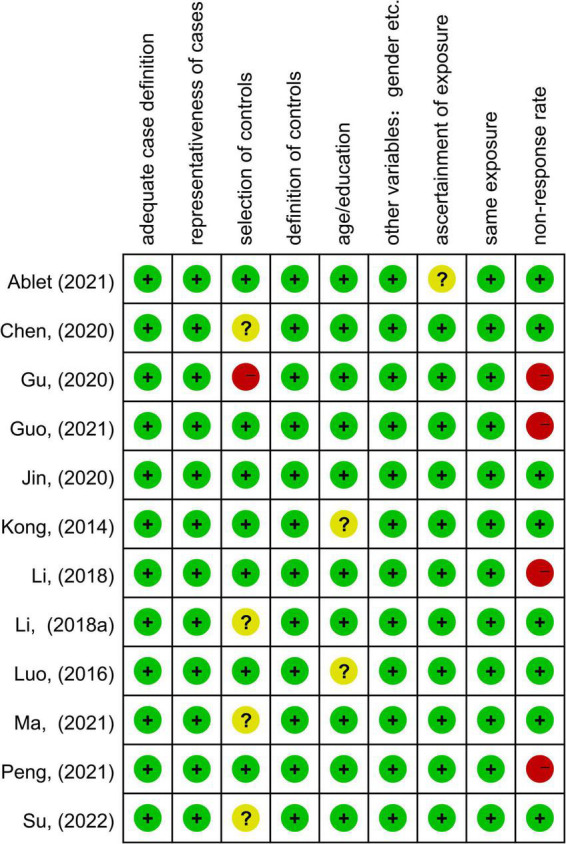
Summary of risk of bias for included studies according to the Newcastle-Ottawa scale (NOS).

Table 1 summarized the demographic, imaging characteristics and quality scores of all studies included in the meta-analysis. ESRD patients and HCs in each of the included studies were matched by age, gender, and education. In this included studies, 345 patients with ESRD (131 females and 214 males, mean age 42.4 years) and 326 HCs (142 females and 184 males, mean age 41.56 years) were reported. All studies underwent whole brain-based ALFF/fALFF analysis (ESRD > HCs: five experiments, 10 Fico; ESRD < HCs: 11 experiments, 90 Foci). In total, 9 of the 11 studies used a 3.0 T scanner for data collection and the remaining two studies were conducted on a 1.5 T scanner. All studies included in the meta-analysis had a quality score of ≥8, indicating that the overall quality of these studies was high. A specific assessment of the risk of bias for each of the included studies is shown in Figure 2.

**TABLE 1:** Demographic and imaging characteristics and quality scores of all studies included in the meta-analysis.


Authors (year of publication)	Method of analysis	*N*	Age in year (SD)	Gender (M/F)	Contrasts	Foci	Tal or MNI	MoCA	Education (years)	Scanner	Software	Quality scores (out of 9)
Kong et al. (2014)	fALFF	ESRD 64	34 (10)	43/21	ESRD < HCs	12	MNI	NA	NA	3.0 T	SPM8	4/1/3
		HCs 45	31 (10)	30/15	ESRD > HCs	0						
Luo et al. (2016)	ALFF	ESRD 24	34 (8)	16/8	ESRD < HCs	8	MNI	NA	NA	3.0 T	SPM8	4/1/3
		HCs 24	32 (9)	16/8	ESRD > HCs	0						
Li et al. (2018)	ALFF	ESRD 23	34 (8.7)	16/7	ESRD < HCs	15	Tal	26.8 (2.7)	11.9 (2.5)	3.0 T	SPM8	4/2/2
		HCs 25	33 (10.3)	17/8	ESRD > HCs	3		23.9 (2.5)	12.9 (3.0)			
Li et al. (2018)	ALFF	ESRD 28	34.1 (8.4)	20/8	ESRD < HCs	16	MNI	23.94 (2.46)	11.93 (2.51)	3.0 T	SPM8	3/2/3
		HCs 25	32.8 (10.4)	18/7	ESRD > HCs	0		26.87 (2.73)	26.87 (2.73)			
Chen et al. (2020)	ALFF	ESRD 19	45.2 (6.7)	6/13	ESRD < HCs	2	MNI	25.0 (3.2)	10.2 (2.7)	3.0 T	DPARSF	3/2/3
		HCs 17	41.8 (9.9)	9/8	ESRD > HCs	2		27.1 (2.4)	12.2 (3.0)			
Jin et al. (2020)	ALFF	ESRD 46	53.11 (1.58)	28/18	ESRD < HCs	2	MNI	NA	NA	3.0 T	DPARSF	4/2/3
		HCs 47	55.57 (0.86)	22/25	ESRD > HCs	2						
Adil et al. (2021)	ALFF	ESRD 24	39.04 (11.84)	15/9	ESRD < HCs	3	MNI	NA	9.71 (3.30)	1.5 T	SPM12	4/2/2
		HCs 20	35.15 (6.07)	7/13	ESRD > HCs	0			11.40 (3.02)			
Guo et al. (2021)	ALFF	ESRD 42	51 (10.2)	22/20	ESRD < HCs	7	MNI	22.5 (4.25)	12 (3)	3.0 T	DPABI	4/2/2
		HCs 42	50 (10.0)	22/20	ESRD > HCs	2		25.5 (4.25)	9 (3)			
Ma et al. (2021)	ALFF/fALFF	ESRD 31	46 (14)	19/12	ESRD < HCs	3	MNI	NA	12.3 (2.7)	3.0 T	REST plus	3/2/3
		HCs 37	43 (12)	20/17	ESRD > HCs	0			13.0 (3.0)			
Peng et al. (2021)	ALFF	ESRD 24	56.21 (11.85)	14/10	ESRD < HCs	20	MNI	NA	11.71 (3.45)	1.5 T	SPM8	4/2/3
		HCs 27	50.74 (11.20)	10/17	ESRD > HCs	0			12.04 (4.00)			
Su et al. (2021)	ALFF	ESRD 20	37.1 (8.6)	15/5	ESRD < HCs	2	MNI	25.4 (1.7)	12.2 (2.8)	3.0 T	DPARSF	3/2/3
		HCs 17	38.5 (6.9)	13/4	ESRD > HCs	1		26.6 (1.5)	13.2 (3.1)			

ESRD, end-stage renal disease; ALFF, amplitude of low-frequency fluctuation; fALFF, fractional amplitude of low-frequency fluctuation; HCs, healthy controls; MNI, Montreal Neurological Institute; Tal, Talairach; MoCA, Montreal Cognitive Assessment; NA, not available; M, male; F, female; SD, standard deviation.

### Meta-analysis results

**FIGURE 3:**
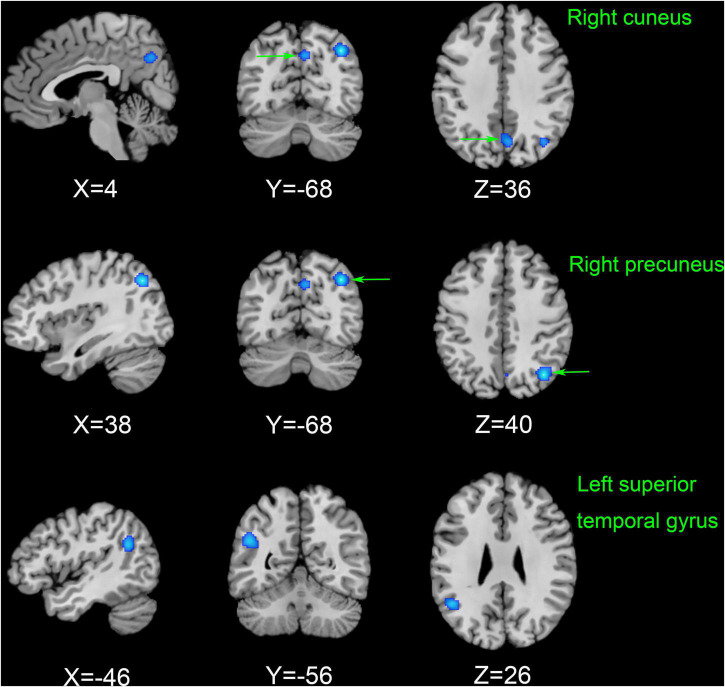
The results of the meta-analysis of all 11 datasets compared the differences between ESRD dialysis patients and HCs. Areas of reduced ALFF/fALFF are shown in blue relative to HCs. The results reached the threshold at *p* < 0.001 cluster-corrected and *p* < 0.05 FWE corrected. ESRD, end-stage renal disease; HCs, healthy controls; ALFF, amplitude of low-frequency fluctuation; fALFF, fractional amplitude of low-frequency fluctuation; FWE, family-wise error.

As shown in Figure 3, according to the results of the ALE meta-analysis, ESRD patients had decreased ALFF/fALFF in the right precuneus (Brodmann area 19, BA 19), right cuneus (BA 7), and left superior temporal gyrus (STG) (BA 39) compared to HCs, while no brain regions with increased ALFF/fALFF were identified. Table 2 showed the coordinates of the cluster maximums.

**TABLE 2:** All clusters from the ALE analysis.

Cluster	Volume (mm^3^)	Coordinates (MNI space)			ALE value	*Z*-value	Side	Anatomical regions	BA
							
		*X*	*Y*	*Z*					
**ESRD < HCs**									
1	1,184	38	−68	40	0.0226	5.596	R	Precuneus	19
2	1,024	−46	−56	26	0.0181	4.921	L	Superior temporal gyrus	39
3	544	4	−68	36	0.0147	4.258	R	Cuneus	7
**ESRD > HCs**									
NA									

ESRD, end-stage renal disease; HCs, healthy controls; MNI, Montreal Neurological Institute; ALE, activation likelihood estimation; BA, Brodmann area; NA, not available.

### Jackknife sensitivity analysis

In Table 3, jackknife sensitivity analysis showed that the brain regions identified by ALE meta-analysis were all highly reliable. Of these, the decreased ALFF/fALFF in the right precuneus was the most reliable and replicable in all 11 datasets. The right cuneus and left STG were also highly replicable, as they were significant in 10 and 8 datasets, respectively.

**TABLE 3:** Jackknife sensitivity analyses.

All studies but…	Right precuneus	Left STG	Right cuneus
Kong et al. (2014)	Yes	Yes	Yes
Luo et al. (2016)	Yes	Yes	Yes
Li et al. (2018)	Yes	Yes	Yes
Li et al. (2018)	Yes	No	Yes
Chen et al. (2020)	Yes	Yes	Yes
Jin et al. (2020)	Yes	Yes	Yes
Adil et al. (2021)	Yes	Yes	No
Guo et al. (2021)	Yes	Yes	Yes
Ma et al. (2021)	Yes	Yes	Yes
Peng et al. (2021)	Yes	Yes	No
Su et al. (2021)	Yes	Yes	No
Total	11 out of 11	10 out of 11	8 out of 11

STG, superior temporal gyrus.

## Discussion

In this study, we used an ALE meta-analysis to explore changes in abnormal neuronal activity in ESRD compared to HCs. To our knowledge, no meta-analysis on such topics has been published. The abnormal brain neuronal activity identified in the meta-analysis mainly involved the default mode network (DMN) (right precuneus), the visual recognition network (VRN) (right cuneus), and the executive control network (ECN) (left STG). Jackknife sensitivity analyses found these results to be highly reliable and not driven by individual studies. Overall, the changes in specific brain regions analyzed in this study are valuable in helping us to uncover possible neural mechanisms of CI in ESRD dialysis patients.

Our meta-analysis showed that ALFF/fALFF was decreased in the right precuneus, right cuneus, and left STG in the patient group compared with HCs. However, no brain regions with increased ALFF/fALFF were found. This is consistent with previous findings from studies of ESRD patients (Luo et al., 2016; Peng et al., 2021). Decreased ALFF/fALFF reflects diminished spontaneous activity of brain neurons and impaired brain function, while increased ALFF/fALFF may be a compensatory mechanism (Yang et al., 2007). A growing number of studies suggested that CI in ESRD patients may be associated with disrupted connectivity in the DMN. The DMN is associated with spontaneous cognitive functions, external environmental monitoring functions, and internal mental activity and is the most widely studied resting-state subnetwork in ESRD patients, including the posterior cingulate cortex, medial prefrontal cortex, precuneus, hippocampus, inferior parietal lobule, and lateral temporal cortex (Raichle and Snyder, 2007; Ni et al., 2014). These regions are usually more active when the brain is in resting state but will deactivate when performing tasks, corresponding to brain regions showing negative activation.

The precuneus, located on the medial aspect of the parietal lobe, is a key node in the DMN and plays a crucial role in various cognitive processes. Functionally, it is closely associated mainly with memory, emotion, and visuospatial executive functions (Bullmore and Sporns, 2009). A graph theory-based analysis pointed out that impaired function of the DMN may underlie CI in ESRD, and further correlation analysis showed that function of the precuneus in ESRD patients correlated with cognitive performance (Yue et al., 2021). This finding was confirmed in the present study. An ASL imaging performed by Cheng et al. (2019) demonstrated a widespread decrease in cerebral blood flow (CBF) in dialysis patients compared to non-dialysis patients. To our knowledge, atrophy of the gray matter cortex of the brain is associated with lower CBF, which can lead to memory deficits and CI (Drew et al., 2013). The study also found a consistent reduction in precuneus ReHo values in ESRD patients and a positive correlation with digit symbol test scores (Liang et al., 2013). This resonates with our study and suggests that decreased ALFF/fALFF in the precuneus may be a potentially promising biomarker for predicting CI in ESRD patients.

The cuneus is located in the occipital lobe of the brain and is an important part of the VRN, with the main functions involving the processing of visual information, facial perception, emotion, and working memory (Parker et al., 2014; He et al., 2021). Abnormal FC in the cuneus has been shown to be associated with altered brain activity in ESRD patients (Su et al., 2021). Previous studies have also shown widespread impairment of the VRN in ESRD dialysis patients (Rozeman et al., 1992). In addition, Peng et al. (2021) showed decreased ALFF in the bilateral cuneus based on rs-fMRI analysis. All of these suggest that dysfunction in the cuneus may cause slowed integration of visual information processing and working memory. However, it is noteworthy that studies using nodal centrality analysis found significant activation of the cuneus in ESRD patients (Wu et al., 2020). These inconsistent results may be related to the severity of the patients’ disease, small sample sizes, and different study methods.

In addition, our meta-analysis found decreased low-frequency brain activity in the left STG. The STG is part of the auditory language center, as well as the ECN. This network is involved in several higher cognitive tasks and has a significant role in attention allocation, goal-directed behavior, and control of emotions (Song et al., 2021). It has been shown that FC in the ECN is associated with substance-dependent approach behavior and that approach behavior is stronger in the left-sided ECN in the resting state (Krmpotich et al., 2013). This is consistent with our findings. In an F-18-fluorodeoxyglucose PET study, Song et al. (2008) found reduced perfusion in the STG and significantly reduced cerebral metabolism in ESRD patients. Using diffusion tensor imaging (DTI), Hsieh et al. (2009) observed lower fractional anisotropy in depression-related regions (STG) in older adults and ESRD dialysis patients than in HCs. To our knowledge, the prevalence of depression among ESRD patients is as high as 12–52% (Cohen et al., 2016). Previous studies have also shown that disruption of FC in the STG is associated with multiple psychological disorders, including depression (Gutman et al., 2009). As we hypothesized, each of these findings could partially explain cognitive dysfunction in ESRD dialysis patients, for example, memory, balance, and emotional processing. Therefore, we presume that a decreased ALFF/fALFF in the STG may be a sign of mental impairment in ESRD dialysis patients.

In this study, we only found positive results for decreased ALFF/fALFF and did not find positive results for increased ALFF/fALFF. However, many single studies have been performed showing that ESRD patients present with common brain abnormalities, such as the increased ALFF/fALFF in the right precentral gyrus (Chen et al., 2020; Su et al., 2021). Yet, no positive results for it were found in our meta-analysis. This may be due to the limitations of the ALE meta-analysis. ALE meta-analysis as a probabilistic analysis can effectively eliminate false positive results and avoid the problem of low statistical test power in individual imaging studies, but it is difficult to avoid false negative results (Radua et al., 2012). In addition, only five trials and 10 foci from our meta-analysis were included in the analysis of increased ALFF/fALFF. A small number of coordinates may not have reached the threshold. These may lead to a decreased accuracy of the results. On the other hand, this may also be related to the influence of confounding factors such as gender, age, and education level of the study.

In the present study, we only analyzed ALFF/fALFF studies in ESRD patients, excluding other imaging studies such as ReHo, DTI, and FC, which to some extent reduces the bias of the results due to the combination of rs-fMRI with other analysis methods. Nevertheless, this study still has some limitations. First, our dataset was limited in number (11 studies) and some results should still be interpreted with caution. Second, our meta-analysis was unable to determine the causal relationship between ESRD and abnormal brain activity. This was due to the fact that the studies we included were cross-sectional studies. Third, the studies included in our meta-analysis were all from Asian countries and may be limited by the application of other national populations. Fourth, owing to insufficient data from relevant studies, we only analyzed ESRD patients on dialysis and did not perform a meta-analysis of dialysis modalities. Finally, because the ALE meta-analysis method did not consider the intensity of activation, it was possible that some brain regions with low activation levels were ignored.

## Conclusion

In summary, using the ALE method, the current meta-analysis shows that in the resting state, ESRD dialysis patients have abnormal spontaneous low-frequency brain activity compared to HCs, mainly involving the DMN, VRN, and ECN. These findings may be potential imaging biomarkers of CI in ESRD dialysis patients and could be considered as a focus of attention for follow-up studies. Meanwhile, more in-depth studies are needed in the future to validate our results and explore more specific biomarkers of early stage of CI in ESRD dialysis patients.

## Data availability statement

The original contributions presented in this study are included in the article/supplementary material, further inquiries can be directed to the corresponding authors.

## Author contributions

HC and FL drafted and revised the manuscript critically for important intellectual content, contributed to the search of the literature, the collection of relevant information, and the data analysis. XF and EZ agreed to be accountable for all aspects of the work in ensuring that questions related to the accuracy or integrity of any part of the work are appropriately investigated and resolved. BK, JS, and YX contributed to the concept and design of the study. All authors read and approved the final manuscript.

## Footnotes

1
http://www.brainmap.org/ale

2
http://ric.uthscsa.edu/mango/

## Abbreviations

ESRD: end-stage renal disease
CI: cognitive impairment
rs-fMRI: resting-state functional magnetic resonance imaging
ALFF: amplitude of low-frequency fluctuation
fALFF: fractional amplitude of low-frequency fluctuation
HCs: healthy controls
ALE: activation likelihood estimation
ASL: arterial spin-labeled
PET: positron emission tomography
BOLD: blood oxygen level-dependent
MNI: Montreal Neurological Institute
FEW: family-wise error
MoCA: Montreal cognitive assessment
ReHo: regional homogeneity
BA: Brodmann area
DMN: default mode network
VRN: visual recognition network
ECN: executive control network
STG: superior temporal gyrus
CBF: cerebral blood flow
FC: functional connectivity
DTI: diffusion tensor imaging.
